# Effectiveness and safety of four drainage methods for lung abscess: a Bayesian network meta-analysis and systematic review

**DOI:** 10.3389/fmed.2025.1735888

**Published:** 2026-01-08

**Authors:** Xuxin Zhang, Yin Zheng, Lijian Pang, Xiaodong Lv, Ningzi Zang

**Affiliations:** 1 Liaoning University of Traditional Chinese Medicine, Shenyang, Liaoning, China; 2 The Affiliated Hospital of Liaoning University of Traditional Chinese Medicine, Shenyang, Liaoning, China

**Keywords:** bayesian model, conventional postural drainage, CT-guided percutaneous drainage, meta-analysis, modified postural drainage, ultrasound-guided percutaneous drainage

## Abstract

**Objective:**

To compare the efficacy and safety of various pus drainage methods for lung abscess via network meta-analysis.

**Methods:**

Randomized controlled trials (RCTs) from PubMed, Embase, Scopus, Web of Science, VIP, Wanfang, and CNKI were searched up to April 2025. Study quality was assessed using the Cochrane Risk of Bias Tool (v5.4.0). Bayesian network meta-analysis was conducted using Rstudio (v4.4.1).

**Results:**

A total of 23 RCTs (1,453 patients) evaluating four drainage techniques were included. Effective rate: CT-guided percutaneous drainage + antibiotics ranked highest (77% probability), outperforming antibiotics alone (OR = 5.4) and conventional postural drainage + antibiotics (OR = 7.4). Hospital stay: Ultrasound-guided drainage + antibiotics ranked best (89% probability), significantly reducing stay versus conventional postural drainage + antibiotics (MD = –19 days). Symptom resolution: Ultrasound-guided drainage + antibiotics ranked highest for cough (SUCRA 97%) and fever resolution (SUCRA 97%). Lesion reduction: Modified postural drainage + antibiotics ranked highest (98% probability), surpassing conventional postural drainage + antibiotics (OR = 0.48) and antibiotics alone (OR = 0.15).

**Conclusion:**

Comprehensive analysis indicates that ultrasound-guided percutaneous drainage combined with antibiotics demonstrates the highest probability of improvement in key outcome measures, including time to fever resolution, length of hospital stay, and time to cough resolution, potentially showcasing a comprehensive potential for clinical benefit. CT-guided percutaneous drainage combined with conventional antibiotic therapy may be the most effective in terms of the effective rate, while modified postural drainage combined with conventional antibiotic therapy may be the most prominent in increasing the number of lesion reduction. Furthermore, conventional antibiotic therapy alone was inferior to pus drainage procedures combined with conventional antibiotics across multiple outcome measures and was associated with a higher incidence of adverse reactions. However, due to the limited number of available studies, these findings can only be considered preliminary. Future validation through a substantial number of multicenter, large-sample, double-blind randomized controlled trials is warranted.

**Systematic review registration:**

https://www.crd.york.ac.uk/PROSPERO/, identifier CRD420251127786.

## 1 Introduction

In clinical respiratory diseases, pulmonary abscess is a suppurative necrotizing disease of lung tissue caused by pathogenic microorganisms, often secondary to aspiration or hematogenous infections (1). Its initial clinical manifestations resemble pneumonia, including high fever and cough; as the condition progresses, characteristic symptoms such as expectoration of large amounts of foul-smelling purulent sputum may appear (2). The disease has an insidious onset and a protracted course. If the infection is not controlled timely and effectively, it can progress to severe complications such as empyema, respiratory failure, bronchopleural fistula, and chest wall fistula (3), potentially even becoming life-threatening. Systemic antibiotic therapy is the standard treatment for pulmonary abscess (4). However, the risk of treatment failure with antibiotics alone is higher in patients with immunocompromised status, comorbid malignancies, advanced age ( > 65 years), impaired consciousness, or specific pathogen infections such as *Klebsiella pneumoniae*, *Pseudomonas aeruginosa*, and *Staphylococcus aureus* (5). Furthermore, for abscesses with a diameter > 6 cm, the response rate to antibiotic therapy is significantly reduced, often necessitating combined drainage treatment. Therefore, although conservative antibiotic therapy is the first-line and gold-standard option, if a patient’s clinical symptoms are not resolved after systemic antibiotic treatment, strategies such as percutaneous, endoscopic drainage, or surgical intervention should be considered for the management of pulmonary abscess (6). Before the advent of antibiotics, the mortality rate of untreated pulmonary abscess was approximately 75%. Compared to the era of antibiotic therapy alone, the addition of percutaneous drainage further reduced the mortality rate of pulmonary abscess to between 20 and 35% (7). Approximately 10% of patients with pulmonary abscess require surgical resection. The efficacy of surgical treatment depends on the patient’s immunity and general physical condition. Some older patients with poor pulmonary function have low tolerance and high risk for thoracotomy (8). Furthermore, the mortality rate for surgical resection is approximately 11–28%, with particularly higher risks in elderly patients, those with multiple comorbidities, or those with complex abscesses (9). Percutaneous pulmonary abscess drainage offers higher safety compared to thoracotomy and better preserves pulmonary function, making it the preferred intervention for patients at high surgical risk. In summary, to shorten the treatment course, reduce medical costs, and improve the cure rate, priority should be given to minimally invasive drainage techniques with fewer complications for timely pus evacuation, combined with effective anti-infective agents for comprehensive treatment (10, 11). Through the review and data analysis of relevant literature, we have summarized the four most commonly used pus drainage methods in the clinical treatment of pulmonary abscess: CT-guided percutaneous drainage, ultrasound-guided percutaneous drainage, conventional postural drainage, and modified postural drainage. Furthermore, traditional systematic reviews and meta-analyses can only perform pairwise comparisons and are unable to conduct quantitative comparisons and ranking of all intervention measures in the absence of direct comparisons. This undoubtedly limits their practicality in guiding clinical decision-making involving multiple options. In contrast, Bayesian network meta-analysis (NMA) compares all interventions through mixed-effects modeling and provides probability rankings. Compared with frequentist methods, Bayesian NMA can more flexibly handle complex models, provide treatment ranking probabilities such as the surface under the cumulative ranking curve (SUCRA) values, and intuitively present the uncertainty of the results. These characteristics make it particularly suitable for providing decision-making references for this clinical question.

## 2 Materials and methods

### 2.1 Databases and search strategy

We conducted an electronic search in the PubMed, Embase, Scopus, Web of Science, VIP, Wanfang, and CNKI databases for randomized controlled trials (RCTs) relevant to this study. The search was performed from database inception to April 2025. The development of the search strategy adhered to the requirements for systematic reviews, focusing on the core concept of pulmonary abscess and the four target interventions along with their synonyms. We employed a combination of Medical Subject Headings (MeSH) terms and abstract, using Boolean operators (AND, OR, NOT) for combination. The searches were tailored to the terminology systems and syntax characteristics of each respective database. The comprehensive search method, using PubMed as an example, is described in Supplementary Table 1.

### 2.2 Inclusion criteria

a. Study type: Randomized controlled trials (RCTs) published before April 2025, comparing different pus drainage procedures for pulmonary abscess versus control treatments for pulmonary abscess, were included regardless of blinding. The design, conduct, and reporting of this study adhere to the Preferred Reporting Items for Systematic Reviews and Meta-Analyses extension for Network Meta-Analysis (PRISMA-NMA) guidelines.

b. Study subjects: Patients with a confirmed diagnosis of pulmonary abscess, meeting the criteria outlined in the Clinical Diagnosis and Treatment Guidelines: Respiratory Medicine Volume (People’s Medical Publishing House, 2009) and the International Classification of Diseases and Related Health Problems 10th Revision (ICD-10) (12, 13). Patients should present with typical clinical manifestations of pulmonary abscess, such as expectoration of foul-smelling purulent sputum, fever, and cough. Pulmonary imaging studies (chest X-ray or CT) should reveal intrapulmonary cavitary lesions or abscess cavities containing air-fluid levels, accompanied by surrounding inflammatory infiltration shadows. Patients were excluded if they had concurrent acute myocardial infarction, severe arrhythmia, massive hemoptysis or active bleeding, or active pulmonary tuberculosis. There were no restrictions on age or gender.

c. Interventions: Comparisons between different drainage methods combined with conventional antibiotics, and comparisons between different drainage methods combined with conventional antibiotics versus conventional antibiotics alone.

d. Outcome measures:

(1) Effective rate (Markedly effective: patient symptoms disappear and laboratory indicators show significant improvement; Improved: patient symptoms show some improvement and laboratory indicators are enhanced to a certain degree; Ineffective: criteria for markedly effective or improved are not met. Total effective rate = (Markedly effective cases + Improved cases)/Total cases × 100%). (2) Length of hospital stay. (3) Time to cough resolution. (4) Time to fever resolution. (5) Number of lesion reduction (assessed via CT and ultrasound examinations to observe the reduction in lesion size).

### 2.3 Exclusion criteria

a. Literature not published in Chinese or English.

b. Study subjects without a clearly confirmed diagnosis of pulmonary abscess meeting the inclusion criteria.

c. Literature with missing, erroneous, or unobtainable (from authors) data on key outcome measures; duplicate publications.

d. Experience summaries, case reports, animal experiments, review articles.

e. Literature that does not specify the type of pus drainage procedure used.

### 2.4 Literature screening and data extraction

EndNote software was employed to remove duplicates and conduct initial screening of the initially retrieved records, integrating search results from different databases. The advantage of this software lies in its ability to efficiently identify and merge duplicate records from various databases, providing a clean and unified data source for subsequent screening. Full texts were then downloaded, and studies not meeting the inclusion criteria were excluded during the secondary screening. If data were missing or other key information was incomplete in the literature, attempts were made to contact the original authors for clarification. The above process was independently completed by two researchers, who extracted data based on a pre-designed form. In case of disagreement, a third researcher was invited to participate in the discussion until a consensus was reached. Ultimately, a total of 23 articles were included. These studies included comparisons of the impact of different drainage techniques on one or more key outcomes in patients with pulmonary abscess, such as the effective rate, length of hospital stay, time to cough resolution, time to fever resolution, and lesion reduction status. Our search strategy and inclusion criteria were designed to screen for literature that provided comparative data on these core variables. Information including the first author, publication year, country, journal name, sample size (including the male-to-female ratio), patient age, interventions, and outcome measures was systematically extracted using an Excel spreadsheet. The flexible structured data management capabilities of Excel facilitated subsequent data verification and transformation for statistical analysis.

### 2.5 Risk of bias assessment and evidence quality evaluation

The methodological quality of the included studies was assessed using the quality evaluation tool recommended by the Cochrane Handbook for Systematic Reviews of Interventions (Version 5.4). The evaluation primarily covered the following domains: random sequence generation, allocation concealment, blinding of participants and personnel, blinding of outcome assessment, incomplete outcome data, selective reporting, and other potential sources of bias. Additionally, we employed the Grading of Recommendations Assessment, Development, and Evaluation (GRADE) system to classify the strength of recommendations for the body of evidence. During the evidence quality evaluation process, five main factors for downgrading were considered (risk of bias, imprecision, inconsistency, indirectness, and publication bias). Based on the analysis of these factors, the quality of evidence was ultimately categorized as high, moderate, low, or very low.

### 2.6 Statistical analysis

Review Manager 5.4 software was used to independently assess the risk of bias for the included studies. Based on a Bayesian framework, a network meta-analysis model was constructed using R software (employing packages such as “gemtc”) to compare and rank the efficacy and safety of the four drainage methods combined with antibiotic therapy for pulmonary abscess. Outcome measures such as the length of hospital stay, time to cough resolution, and time to fever resolution in this study are continuous variables, expressed as the mean difference (MD) and its 95% credible interval (95% CrI). Outcomes such as the effective rate and the number of lesion reduction in this study are dichotomous variables, expressed as the odds ratio (OR) or risk ratio (RR) and their respective 95% CrI. A difference is generally considered statistically significant if the 95% CrI for a continuous variable does not include 0, or if the 95% CrI for a dichotomous variable does not include 1. Heterogeneity among studies was assessed using the I^2^ statistic, with I^2^ ≥ 50% indicating significant heterogeneity. During model construction, four Markov chains were specified, each assigned different initial values. A burn-in period of 5,000 iterations was set to eliminate the influence of initial values, followed by 20,000 effective iterations. The gelman.diag() function was used to calculate the R-hat value (Potential Scale Reduction Factor, PSRF; now referred to as R-hat) to assess model convergence. A value close to 1.0 indicates good convergence; otherwise, the number of iterations was extended to 50,000 and re-evaluated. Random and fixed effects models were selected based on the deviance information criterion (DIC). DIC values for both random and fixed effects models were calculated, and generally, the model with the smaller DIC value was chosen as the optimal one. If closed loops existed in the network, the node-splitting method was employed to examine inconsistency between direct and indirect evidence (14). For outcomes such as the effective rate that included a sufficient number of studies, small-study effects were assessed by examining funnel plots. Finally, interventions were ranked using the surface under the cumulative ranking curve (SUCRA). A higher SUCRA percentage typically indicates a better treatment effect.

## 3 Results

### 3.1 Literature search results

**FIGURE 1:**
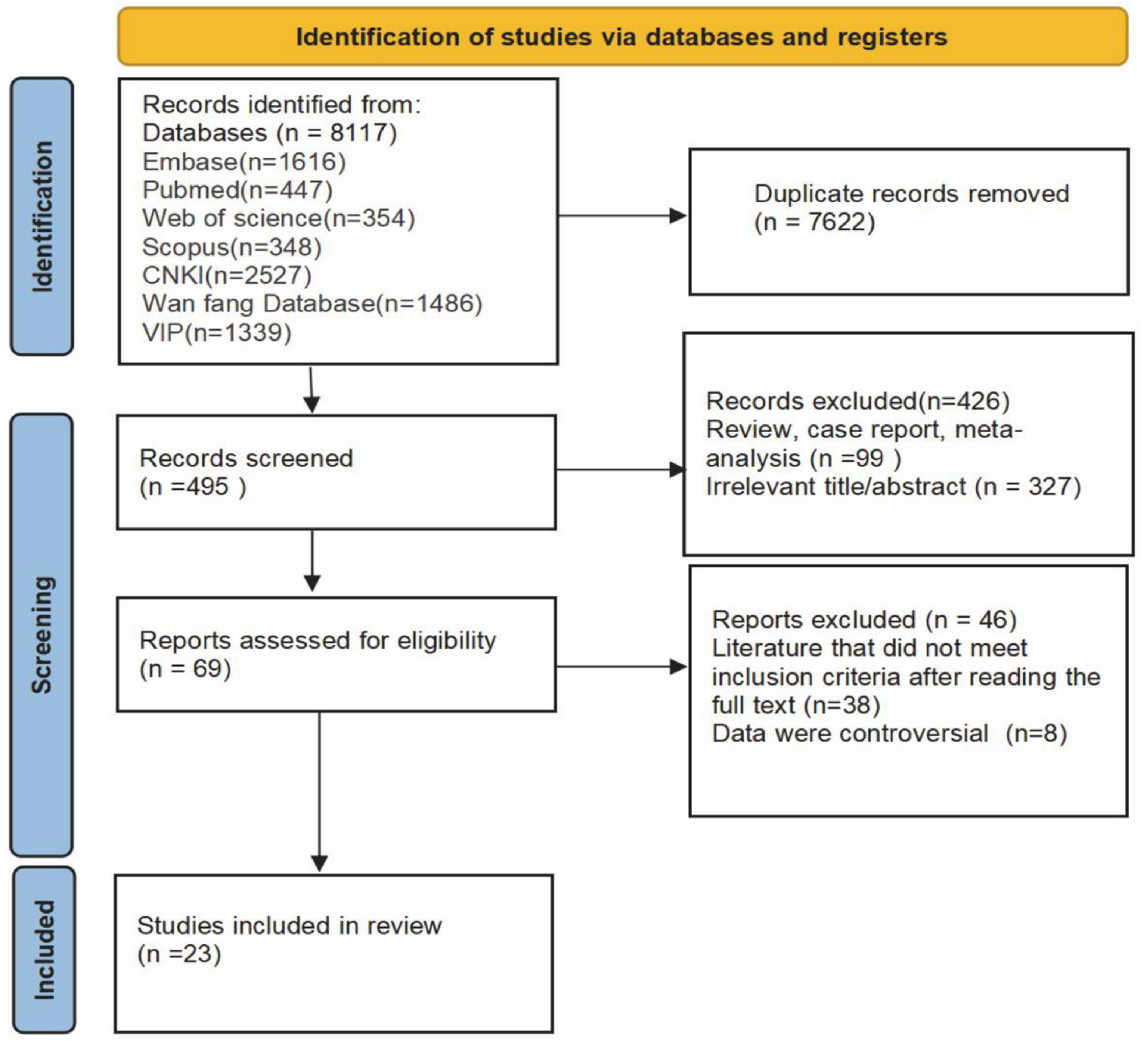
PRISMA flow chart.

A total of 8,117 records were initially retrieved. All records were imported into EndNote software, and 7,622 duplicates were removed. After reviewing titles and abstracts, 426 records were excluded. Following a full-text review, 38 records were excluded for not meeting the inclusion criteria, and an additional 8 records were excluded due to being controversial or not explicitly mentioning the type of pus drainage procedure. Finally, 23 articles were included (15–37), comprising 22 Chinese articles (15–36), and 1 English article (37). The literature screening process is shown in Figure 1.

### 3.2 Characteristics of included studies, risk of bias, and evidence grade assessment

**FIGURE 2:**
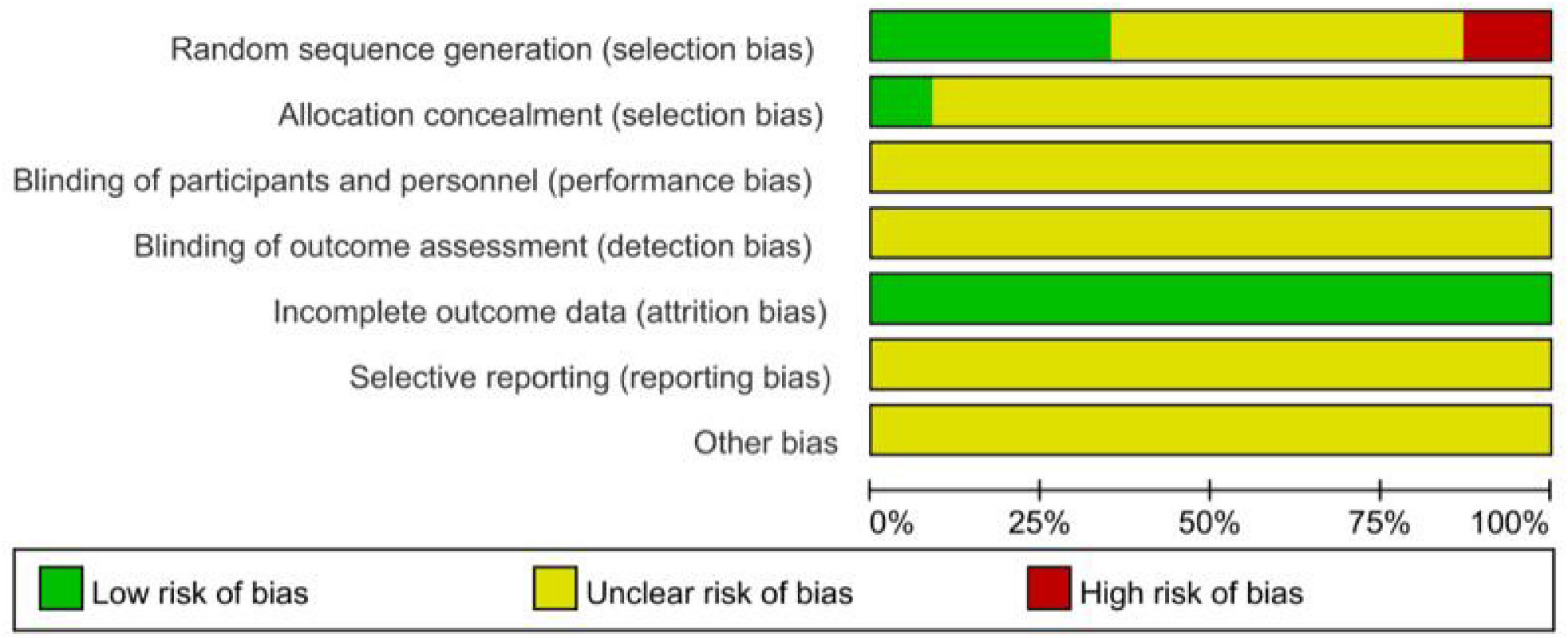
Risk of bias assessment proportion plot.

A total of 23 studies were included (15–37), involving an aggregate sample size of 1,453 patients. Fourteen studies reported the effective rate (16–25, 30, 34, 36, 37), 10 studies reported the length of hospital stay (15, 17, 19, 20, 23–25, 30, 36, 37), nine studies reported the time to cough resolution (15, 19–21, 23–25, 30, 36), nine studies reported the time to fever resolution (15, 19–21, 23–25, 30, 36), and nine studies reported the number of lesion reduction (26–29, 31–33, 35, 37). Regarding risk of bias, 12 studies specified the randomization method (17–20, 26, 28, 30, 33–37). Among these, six used a random number table (17, 20, 28, 30, 33, 37); two were randomized by order of admission (18, 36) and one by order of consultation (19), which were considered high risk; and two used sealed envelope drawing (26, 35). None of the included studies described blinding in outcome assessment and were therefore judged as having unclear risk. The risk for incomplete outcome data was low, while risks for other biases and selective reporting were unclear. The basic characteristics of the included studies are presented in Table 1, and the risk of bias assessment is shown in Figure 2. We also conducted a GRADE assessment for the five main outcomes across all intervention comparisons; detailed results are provided in Supplementary Table 2. Overall, the quality of evidence for most comparisons was moderate or low. The primary reasons for downgrading included: (1) Risk of bias—most studies had high or unclear risk regarding blinding and randomization, which particularly affected the assessment of subjective outcomes like cough and fever resolution. (2) Imprecision—for some comparisons, the 95% credible intervals were wide and crossed the line of no effect, indicating imprecise estimates. These studies provide the data foundation for subsequent comparisons of the efficacy and safety of different drainage techniques.

**TABLE 1:** Basic characteristics of the included studies.

Author (year)	Country	N(M/F)		Average age/year		Intervention(s)		Outcome measure(s)
		T	C	T	C	T	C	
Huang J 2023 (15)	CHN	16/14	19/11	49.69 ± 1.45	49.65 ± 1.46	Linezolid combined with CT-guided abscess drainage	Linezolid	➁➂➃
Zhang HY 2023 (16)	CHN	22/14	21/15	39.56 ± 4.23	39.48 ± 4.31	Modified postural drainage combined with conventional antibiotic therapy	Conventional drainage combined with conventional antibiotic therapy	➀
Shen JM 2019 (17)	CHN	23/13	22/14	47.5 ± 5.6	47.1 ± 6.0	Linezolid combined with CT-guided abscess drainage	Linezolid	➀➁
Wu SL 2018 (18)	CHN	57 /25		42.58 ± 5.23		Moxifloxacin combined with clindamycin administration therapy combined with CT-guided abscess drainage	Moxifloxacin combined with clindamycin therapy	➀
Yan YJ 2018 (19)	CHN	23/17		48.63 ± 4.28		Linezolid combined with CT-guided abscess drainage	Linezolid	➀➁➂➃
Fang TJ 2014 (20)	CHN	11/8	10/9	48.2 ± 6.7	48.1 ± 5.3	Ultrasound-guided percutaneous drainage combined with conventional antibiotic therapy	Conventional postural drainage combined with conventional antibiotic therapy	➀➁➂➃
Li WJ 2013 (21)	CHN	13/17	16/14	46 ± 2.3	41 ± 4.5	Ultrasound-guided percutaneous drainage combined with conventional antibiotic therapy	Conventional postural drainage combined with conventional antibiotic therapy	➀➂➃
Qi W 2011 (22)	CHN	20/12	18/14	43	45	CT-guided abscess drainage combined with conventional antibiotic therapy	Conventional antibiotic therapy	➀
Zhang S 2010 (23)	CHN	26/9	24/8	49.6 ± 16.62	50.31 ± 17.32	CT-guided abscess drainage combined with conventional antibiotic therapy	Conventional postural drainage combined with conventional antibiotic therapy	➀➁➂➃
Fu P 2007 (24)	CHN	24/10	22/8	48.3	46.7	Ultrasound-guided percutaneous drainage combined with conventional antibiotic therapy	Conventional postural drainage combined with conventional antibiotic therapy	➀➁➂➃
Chen GH 2015 (25)	CHN	24/13	20/15	48.3	47.8	CT-guided abscess drainage combined with conventional antibiotic therapy	Conventional postural drainage combined with conventional antibiotic therapy	➀➁➂➃
Wang Y 2016 (26)	CHN	34/16		42.3 ± 5.4		Modified postural drainage combined with conventional antibiotic therapy	Conventional postural drainage combined with conventional antibiotic therapy	➄
Jia K 2010 (27)	CHN	39/27		53.4		Modified postural drainage combined with conventional antibiotic therapy	Conventional postural drainage combined with conventional antibiotic therapy	➄
Jin D 2016 (28)	CHN	39/27		53 ± 2.4		Modified postural drainage combined with conventional antibiotic therapy	Conventional antibiotic therapy	➄
Chen XQ 2015 (29)	CHN	26/9	28/7	50 ± 4	50 ± 3	Modified postural drainage combined with conventional antibiotic therapy	Conventional postural drainage combined with conventional antibiotic therapy	➄
Liu Y 2016 (30)	CHN	21/18	24/15	44 ± 5	44 ± 5	CT-guided abscess drainage combined with conventional antibiotic therapy	Linezolid	➀➁➂➃
Xu P 2018 (31)	CHN	23/7	21/9	46.5 ± 4.8	45.3 ± 5.6	Modified postural drainage combined with conventional antibiotic therapy	Conventional postural drainage combined with conventional antibiotic therapy	➄
Tian LJ 2016 (32)	CHN	17/8	15/10	52.05 ± 5.11	50.05 ± 4.08	Modified postural drainage combined with conventional antibiotic therapy	Conventional postural drainage combined with conventional antibiotic therapy	➄
Liu JM 2017 (33)	CHN	38/12	35/15	48.21 ± 8.26	48.05 ± 8.12	Modified postural drainage combined with conventional antibiotic therapy	Conventional postural drainage combined with conventional antibiotic therapy	➄
Ding LM 2017 (34)	CHN	14/15	16/13	42.35 ± 5.23	44.36 ± 5.12	Modified postural drainage combined with conventional antibiotic therapy	Conventional postural drainage combined with conventional antibiotic therapy	➀
Yang GY 2019 (35)	CHN	27/4	26/5	42.52 ± 3.43	41.48 ± 3.22	Modified postural drainage combined with conventional antibiotic therapy	Conventional postural drainage combined with conventional antibiotic therapy	➄
Wu ZY 2022 (36)	CHN	24/14	26/12	69.91 ± 1.52	69.56 ± 1.52	Ultrasound-guided percutaneous drainage combined with conventional antibiotic therapy	Conventional postural drainage combined with conventional antibiotic therapy	➀➁➂➃
Mohamed A S 2014 (37)	EGY	7/6	8/5	55.7 ± 8.8	56.5 ± 9.3	Ultrasound-guided percutaneous drainage combined with conventional antibiotic therapy	Conventional antibiotic therapy	➀➁➄

N, total sample size; M, male; F, female; T, trial group; C, control group; Outcome measures: ➀ Effective rate; ➁ Length of hospital stay; ➂ Time to cough resolution; ➃ Time to fever resolution; ➄ Number of lesion reduction.

### 3.3 Convergence assessment and evidence network

**FIGURE 3:**
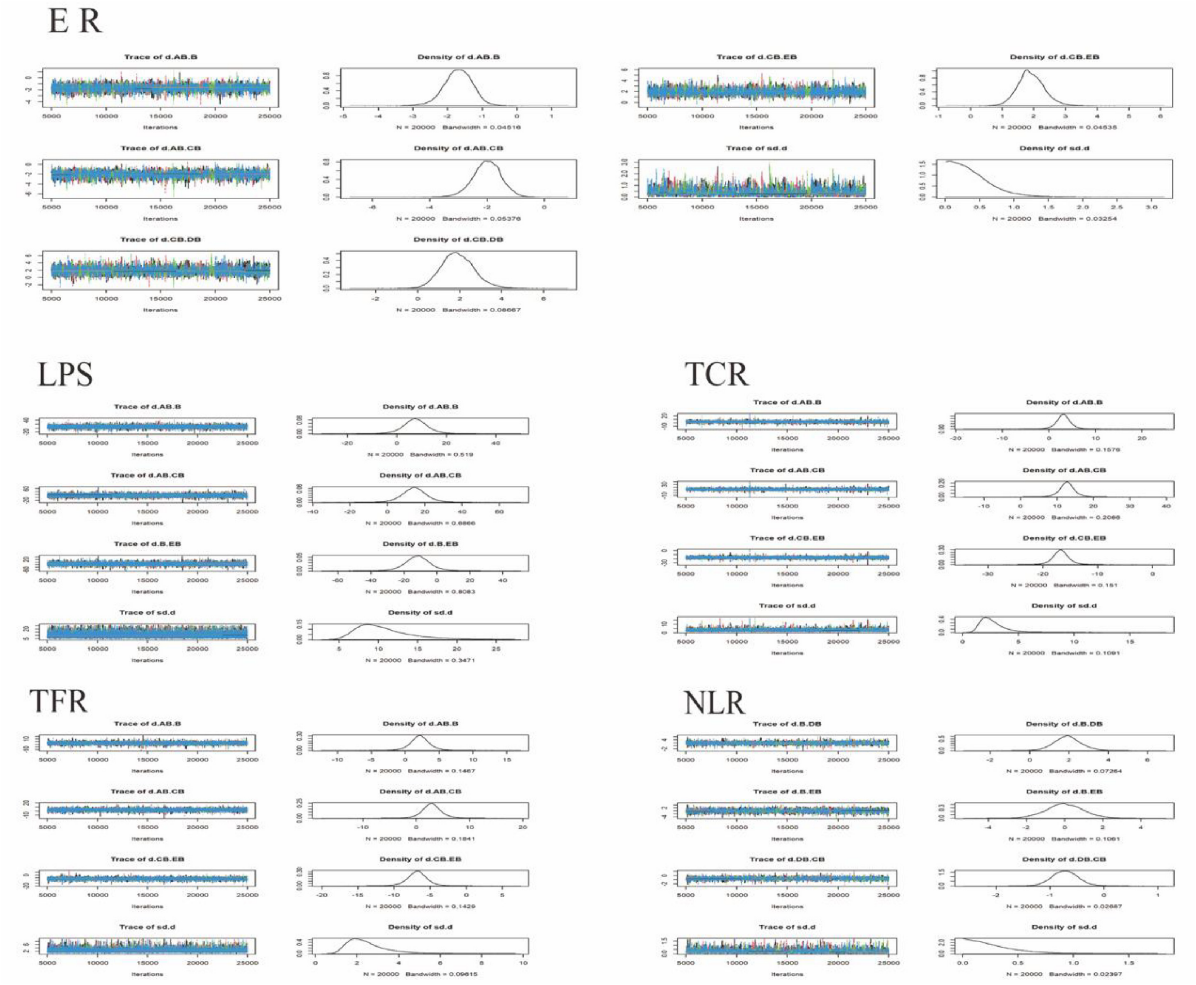
Trace plot and density plot for each outcome indicator. Trace Plots: The *x*-axis represents the number of iterations, and the *y*-axis represents the range of parameter values. The multi-colored curves represent the paths of independent Markov chains, reflecting the exploration of the parameter space and convergence. Density Plots: The *x*-axis represents the possible values of the parameter. The *y*-axis represents the probability density. The total area under the curve integrates to 1. A higher and narrower peak indicates a higher probability of the parameter values in that region. Intervention Codes: AB, CT-guided percutaneous drainage combined with conventional antibiotic therapy CB, Conventional postural drainage combined with conventional antibiotic therapy DB, Modified postural drainage combined with conventional antibiotic therapy EB, Ultrasound-guided percutaneous drainage combined with conventional antibiotic therapy B, Conventional antibiotic therapy alone. ER, Effective Rate; LPS, Length of Hospital Stay; TCR, Time to Cough Resolution; TFR, Time to Fever Resolution; NLR, Number of Lesion Reduction.

**FIGURE 4:**
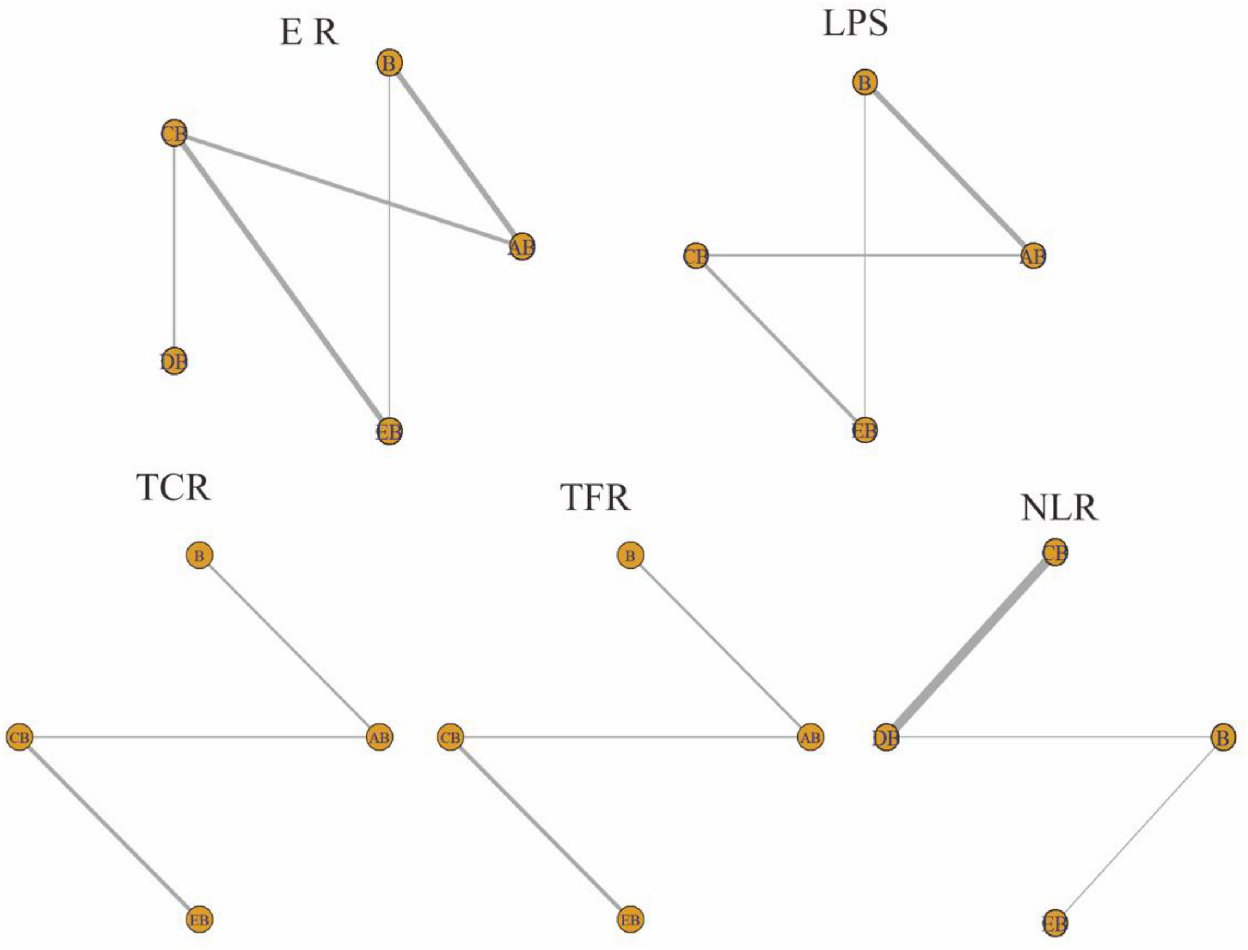
Evidence network for each outcome indicator. Circles represent individual interventions, with their size proportional to the sample size. The thickness of the connecting lines represents the number of studies comparing two interventions. A line between two points indicates a direct comparison between those interventions. Intervention Codes: AB, CT-guided percutaneous drainage combined with conventional antibiotic therapy CB, Conventional postural drainage combined with conventional antibiotic therapy DB, Modified postural drainage combined with conventional antibiotic therapy EB, Ultrasound-guided percutaneous drainage combined with conventional antibiotic therapy B, Conventional antibiotic therapy alone. ER, Effective Rate; LPS, Length of Hospital Stay; TCR, Time to Cough Resolution; TFR, Time to Fever Resolution; NLR, Number of Lesion Reduction.

To compare the four drainage methods, we constructed models. The convergence assessment results are presented in Table 2 and Figure 3, and the evidence networks for each outcome are shown in Figure 4. All parameters demonstrated good convergence, ensuring the reliability of subsequent comparative results. Specifically, the trace plots (maximum iterations: 25,000) showed stable trajectories for most parameters, indicating that the Markov chains achieved good convergence after numerous iterations, with estimates tending to stabilize and the simulation results being highly reliable. The density plots predominantly exhibited unimodal distributions; unimodality suggests a central tendency in the parameter values, with the peak corresponding to the most probable value. All Potential Scale Reduction Factor (PSRF) values were < 1.05, indicating favorable convergence and stability. The included studies involved the following interventions: AB (CT-guided percutaneous drainage combined with conventional antibiotic therapy), CB (conventional postural drainage combined with conventional antibiotic therapy), DB (modified postural drainage combined with conventional antibiotic therapy), EB (ultrasound-guided percutaneous drainage combined with conventional antibiotic therapy), and B (conventional antibiotic therapy alone). Figure 4 presents the evidence network graphs. Closed loops were formed for both the effective rate and the length of hospital stay, allowing for the assessment of inconsistency within these networks.

**TABLE 2:** Potential scale reduction factors.

Parameter	Point estimation	Upper 95% CrI
**ER**		
d. AB. B	1.01	1.02
d. AB. CB	1.00	1.01
d. CB. DB	1.00	1.01
d. CB. EB	1.00	1.00
sd. d	1.01	1.02
**LPS**		
d. AB. B	1.00	1.00
d. AB. CB	1.00	1.00
d. B. EB	1.00	1.00
sd. d	1.00	1.00
**TCR**		
d. AB. B	1.00	1.00
d. AB. CB	1.00	1.00
d. CB. EB	1.00	1.00
sd. d	1.00	1.00
**TFR**		
d. AB. B	1.00	1.00
d. AB. CB	1.00	1.00
d. CB. EB	1.00	1.00
sd. d	1.00	1.00
**NLR**		
d. B. DB	1.00	1.00
d. B. EB	1.00	1.01
d. DB. CB	1.00	1.01
sd. d	1.00	1.01

For each comparison group, the point estimate and upper 95% CrI values are equal to or close to 1, indicating a high degree of convergence in the study results. demonstrating excellent. AB, CT-guided percutaneous drainage combined with conventional antibiotic therapy CB, Conventional postural drainage combined with conventional antibiotic therapy DB, Modified postural drainage combined with conventional antibiotic therapy EB, Ultrasound-guided percutaneous drainage combined with conventional antibiotic therapy B, Conventional antibiotic therapy alone. ER, Effective Rate; LPS, Length of Hospital Stay; TCR, Time to Cough Resolution; TFR, Time to Fever Resolution; NLR, Number of Lesion Reduction.

### 3.4 Consistency assessment

**FIGURE 5:**
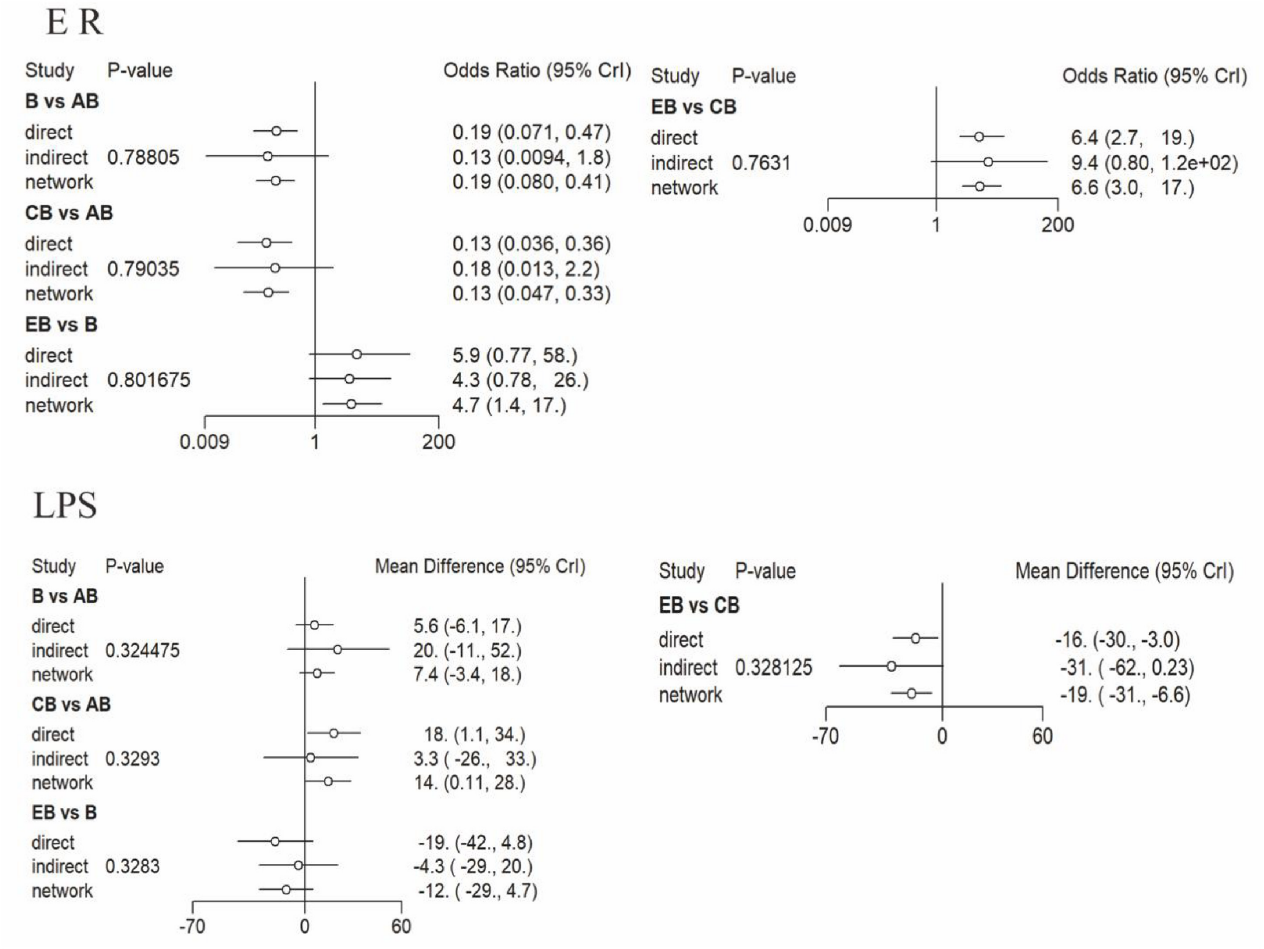
Forest plot for the consistency test. For each comparison, the three points represent the effect estimates from the direct, indirect, and network meta-analysis, respectively. The horizontal lines represent the 95% CrI. Intervention Codes: AB, CT-guided percutaneous drainage combined with conventional antibiotic therapy CB, Conventional postural drainage combined with conventional antibiotic therapy DB, Modified postural drainage combined with conventional antibiotic therapy EB, Ultrasound-guided percutaneous drainage combined with conventional antibiotic therapy B, Conventional antibiotic therapy alone. ER, Effective Rate; LPS, Length of Hospital Stay.

After ensuring model reliability, we conducted inconsistency tests for outcomes with closed loops in the network. The results (Figure 5) indicated consistency between direct and indirect evidence (*P* > 0.05), supporting the use of a consistency model for pooled analysis. From the network graphs, it is evident that both the effective rate and length of hospital stay formed closed loops. Therefore, node-splitting analysis was employed to examine the consistency between direct and indirect comparison results within these loops. Figure 5 presents the inconsistency test results, showing that the effect estimates from direct and indirect comparisons were generally aligned in direction, with *P* > 0.05 indicating no significant inconsistency. This confirms the reliability of the comparison results and justifies the use of a consistency model for analysis.

### 3.5 Heterogeneity and model selection for each outcome

Based on the aforementioned results, Table 3 summarizes the goodness-of-fit statistics for the consistency models under both fixed-effect and random-effects assumptions for each outcome, including the deviance information criterion (DIC), pD, Dbar, and the Icriterion (DIC)ncy models under both fixed-effect and random-effects assumptions for each outcome, including the stency modeuse in subsequent analyses.

**TABLE 3:** Goodness-of-fit and heterogeneity assessment results for the consistency model under different outcome measures.

Outcome measures	Model type	DIC	Dbar	pD	I^2^	Model selection
ER	Random effects	46.50554	26.30504	20.20050	0%	Fixed effects model
	Fixed effects	45.06245	26.76648	18.29587	0%	
LPS	Random effects	40.08199	20.13381	19.94817	6%	Random effects model
	Fixed effects	297.24930	284.15303	13.09626	93%	
TCR	Random effects	35.75073	18.30119	17.44953	7%	Random effects model
	Fixed effects	58.52069	46.54446	11.97623	63%	
TFR	Random effects	35.44091	17.98095	17.45996	5%	Random effects model
	Fixed effects	85.25205	73.14161	12.11044	77%	
NLR	Random Effects	28.70821	15.04385	13.66436	0%	Fixed effects model
	Fixed effects	26.69570	14.48117	12.21453	0%	

DIC, deviance information criterion; Dbar, posterior mean of the deviance; pD, effective number of parameters; I^2^, heterogeneity. ER, Effective Rate; LPS, Length of Hospital Stay; TCR, Time to Cough Resolution; TFR, Time to Fever Resolution; NLR, Number of Lesion Reduction.

### 3.6 Network meta-analysis results

#### 3.6.1 Effective rate analysis results

**FIGURE 6:**
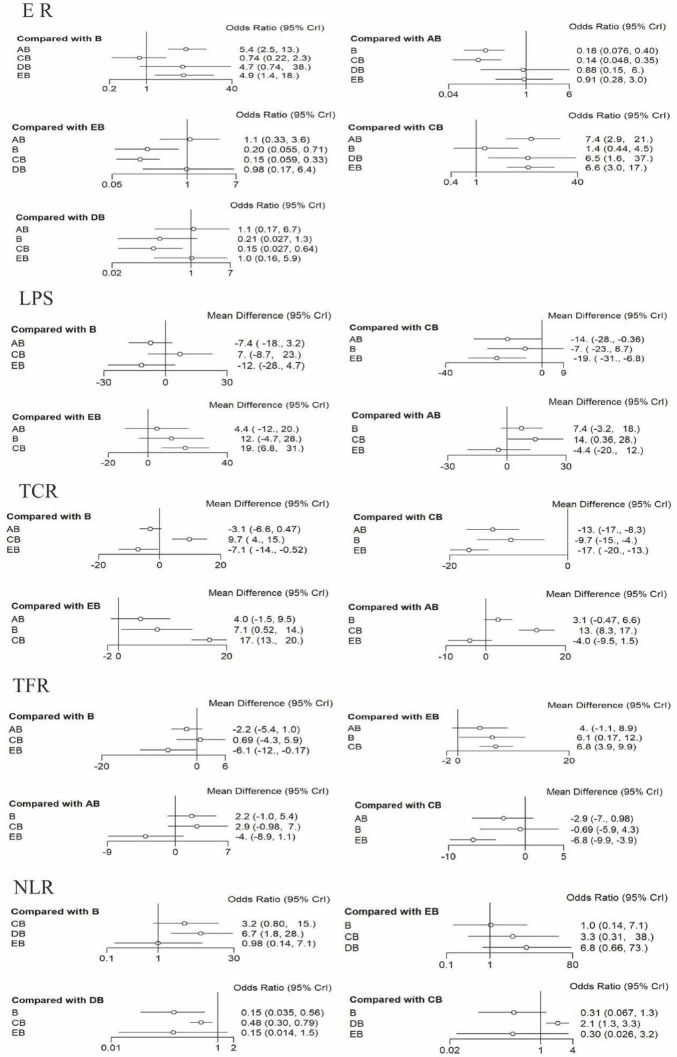
Forest Plots for Each Outcome Indicator. Each point represents the OR or MD of an intervention. The horizontal lines represent the 95% CrI. Intervention Codes: AB, CT-guided percutaneous drainage combined with conventional antibiotic therapy CB, Conventional postural drainage combined with conventional antibiotic therapy DB, Modified postural drainage combined with conventional antibiotic therapy EB, Ultrasound-guided percutaneous drainage combined with conventional antibiotic therapy B, Conventional antibiotic therapy alone. ER, Effective Rate; LPS, Length of Hospital Stay; TCR, Time to Cough Resolution; TFR, Time to Fever Resolution; NLR, Number of Lesion Reduction.

**FIGURE 7:**
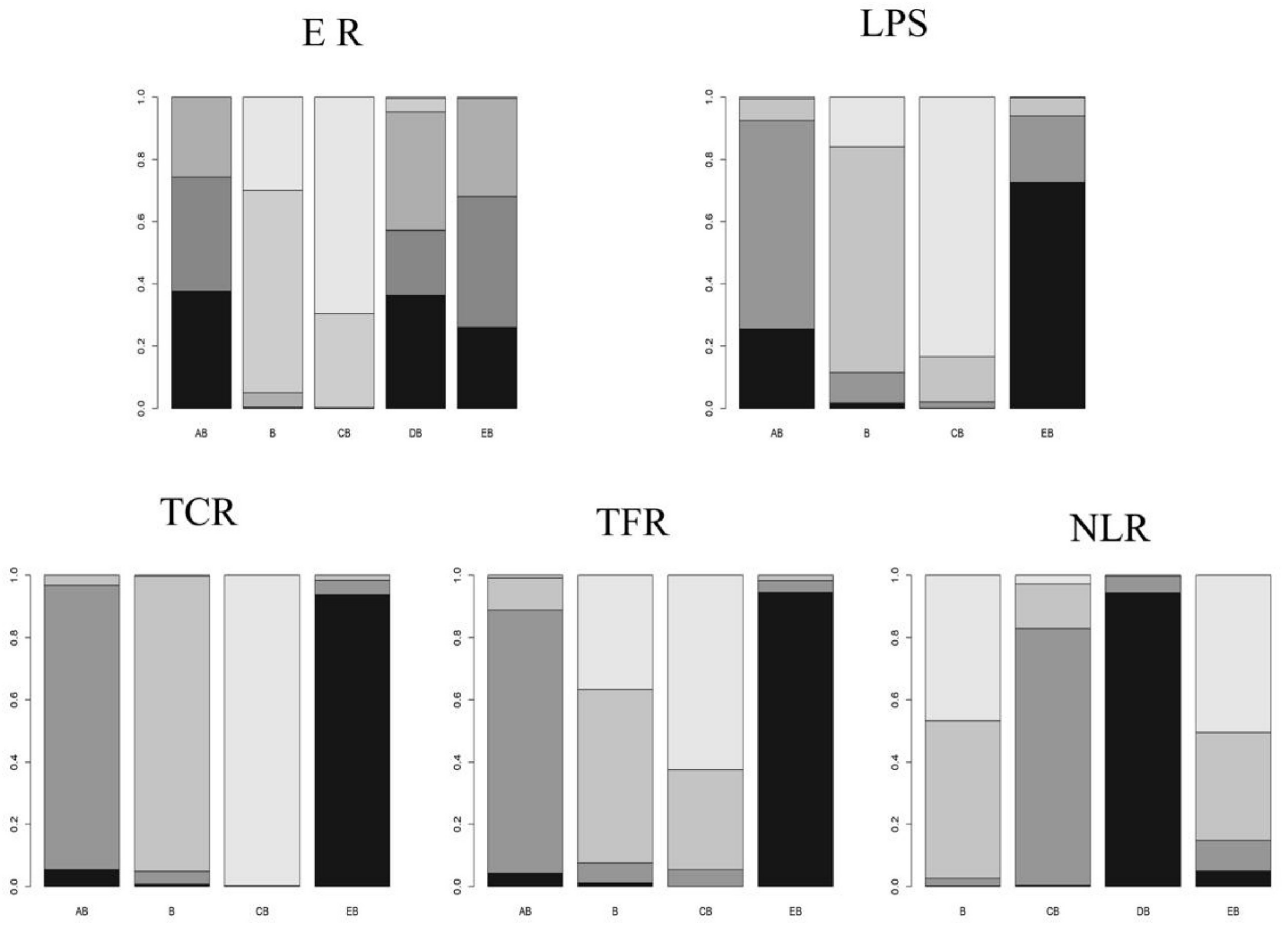
Comparison of the Effects of Different Interventions on Different Outcome Indicators. Different letters on the *x*-axis represent different intervention measures. The *y*-axis represents the cumulative probability (SUCRA value) that each intervention measure ranks as the best for the corresponding outcome indicator. Intervention Codes: AB, CT-guided percutaneous drainage combined with conventional antibiotic therapy CB, Conventional postural drainage combined with conventional antibiotic therapy DB, Modified postural drainage combined with conventional antibiotic therapy EB, Ultrasound-guided percutaneous drainage combined with conventional antibiotic therapy B, Conventional antibiotic therapy alone. ER, Effective Rate; LPS, Length of Hospital Stay; TCR, Time to Cough Resolution; TFR, Time to Fever Resolution; NLR, Number of Lesion Reduction.

Based on the stable evidence network established above, Bayesian network meta-analysis was performed. Fourteen studies reported the effective rate (16–25, 30, 34, 36, 37). Analysis of the effective rate showed that, compared with CT-guided percutaneous drainage combined with conventional antibiotic therapy, conventional antibiotic therapy alone (OR = 5.4; 95% CrI = 2.5, 13) and conventional postural drainage combined with conventional antibiotic therapy (OR = 7.4; 95% CrI = 2.9, 21) were less effective. Compared with ultrasound-guided percutaneous drainage combined with conventional antibiotic therapy, conventional antibiotic therapy alone (OR = 4.9; 95% CrI = 1.4, 18) and conventional postural drainage combined with conventional antibiotic therapy (OR = 6.6; 95% CrI = 3.0, 17) were less effective. Compared with modified postural drainage combined with conventional antibiotic therapy, conventional postural drainage combined with conventional antibiotic therapy (OR = 6.5; 95% CrI = 1.6, 37) was less effective. No statistically significant differences were observed among other intervention comparisons (see Figure 6). According to the surface under the cumulative ranking curve (SUCRA) results, CT-guided percutaneous drainage combined with conventional antibiotic therapy had the highest probability of being effective (77%), followed by ultrasound-guided percutaneous drainage combined with conventional antibiotic therapy (73%), modified postural drainage combined with conventional antibiotic therapy (72%), conventional antibiotic therapy alone (18%), and conventional postural drainage combined with conventional antibiotic therapy (7%) (see Figure 7 and Table 4 for details).

**TABLE 4:** Comprehensive best probability ranking table.

Outcome measures	AB	B	CB	DB	EB
ER	0. 77989062	0. 18852500	0. 07663125	0. 72101875	0. 73393438
LPS	0. 72471667	0. 32433333	0. 06305417	–	0. 88789583
TCR	0. 674112500	0. 351095833	0. 001379167	–	0. 973412500
TFR	0. 6410833	0. 2399667	0. 1434708	–	0. 9754792
NLR	–	0. 1876167	0. 6013250	0. 9798667	0. 2311917

“–” indicates that the intervention was excluded from this metric. Each value represents the overall probability for the corresponding intervention in this specific metric. AB, CT-guided percutaneous drainage combined with conventional antibiotic therapy CB, Conventional postural drainage combined with conventional antibiotic therapy DB, Modified postural drainage combined with conventional antibiotic therapy. EB, Ultrasound-guided percutaneous drainage combined with conventional antibiotic therapy B, Conventional antibiotic therapy alone. ER, Effective Rate; LPS, Length of Hospital Stay; TCR, Time to Cough Resolution; TFR, Time to Fever Resolution; NLR, Number of Lesion Reduction.

#### 3.6.2 Length of hospital stay analysis results

Ten studies reported the length of hospital stay (15, 17, 19, 20, 23–25, 30, 36, 37). Analysis of the length of hospital stay showed that, compared with conventional postural drainage combined with conventional antibiotic therapy, ultrasound-guided percutaneous drainage combined with conventional antibiotic therapy (MD = –19; 95% CrI = –31, –6.8) and CT-guided percutaneous drainage combined with conventional antibiotic therapy (MD = –14; 95% CrI = –28, –0.36) were superior. No statistically significant differences were observed among other comparisons (see Figure 6). According to the surface under the cumulative ranking curve (SUCRA) results, ultrasound-guided percutaneous drainage combined with conventional antibiotic therapy had the highest probability of being the best for reducing hospital stay (89%), followed by CT-guided percutaneous drainage combined with conventional antibiotic therapy (72%), conventional antibiotic therapy alone (32%), and conventional postural drainage combined with conventional antibiotic therapy (6%) (see Figure 7 and Table 4 for details).

#### 3.6.3 Time to cough resolution analysis results

Nine studies reported the time to cough resolution (15, 19–21, 23–25, 30, 36). Analysis of the time to cough resolution showed that, compared with ultrasound-guided percutaneous drainage combined with conventional antibiotic therapy, conventional antibiotic therapy alone (MD = 7.1; 95% CrI = 0.52, 14) was less effective. Compared with conventional postural drainage combined with conventional antibiotic therapy, CT-guided percutaneous drainage combined with conventional antibiotic therapy (MD = –13; 95% CrI = –17, –8.3), conventional antibiotic therapy alone (MD = –9.7; 95% CrI = –15, –4), and ultrasound-guided percutaneous drainage combined with conventional antibiotic therapy (MD = –17; 95% CrI = –20, –13) were superior. No statistically significant differences were observed among the remaining comparisons (see Figure 6). According to the surface under the cumulative ranking curve (SUCRA) results, ultrasound-guided percutaneous drainage combined with conventional antibiotic therapy had the highest probability of being the best for reducing the time to cough resolution (97%), followed by CT-guided percutaneous drainage combined with conventional antibiotic therapy (67%), conventional antibiotic therapy alone (35%), and conventional postural drainage combined with conventional antibiotic therapy (0%) (see Figure 7 and Table 4 for details).

#### 3.6.4 Time to fever resolution analysis results

Nine studies reported the time to fever resolution (15, 19–21, 23–25, 30, 36). Analysis of the time to fever resolution showed that, compared with ultrasound-guided percutaneous drainage combined with conventional antibiotic therapy, conventional antibiotic therapy alone (MD = 6.1; 95% CrI = 0.17, 12) and conventional postural drainage combined with conventional antibiotic therapy (MD = 6.8; 95% CrI = 3.9, 9.9) were less effective. No statistically significant differences were observed among the remaining comparisons (see Figure 6). According to the surface under the cumulative ranking curve (SUCRA) results, ultrasound-guided percutaneous drainage combined with conventional antibiotic therapy had the highest probability of being the best for reducing the time to fever resolution (97%), followed by CT-guided percutaneous drainage combined with conventional antibiotic therapy (64%), conventional antibiotic therapy alone (23%), and conventional postural drainage combined with conventional antibiotic therapy (14%) (see Figure 7 and Table 4 for details).

#### 3.6.5 Analysis results for the number of lesion reduction

Nine studies reported the number of lesion reduction (26–29, 31–33, 35, 37). Analysis of the number of lesion reduction showed that, compared with modified postural drainage combined with conventional antibiotic therapy, conventional postural drainage combined with conventional antibiotic therapy (OR = 0.48; 95% CrI = 0.30, 0.79) and conventional antibiotic therapy alone (OR = 0.15; 95% CrI = 0.035, 0.56) were less effective. No statistically significant differences were observed among the remaining comparisons (see Figure 6). According to the surface under the cumulative ranking curve (SUCRA) results, modified postural drainage combined with conventional antibiotic therapy had the highest probability of being the best for increasing the number of lesion reduction (98%), followed by conventional postural drainage combined with conventional antibiotic therapy (60%), ultrasound-guided percutaneous drainage combined with conventional antibiotic therapy (23%), and conventional antibiotic therapy alone (19%) (see Figure 7 and Table 4 for details).

### 3.7 Publication bias assessment

**FIGURE 8:**
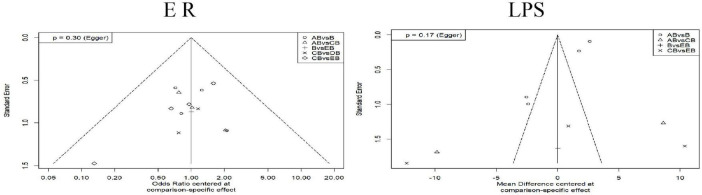
Corrected funnel plot for each outcome indicator. The *x*-axis represents the mean difference based on specific comparisons, reflecting the effect size differences between different interventions. The *y*-axis represents the standard error, indicating the precision of the effect estimate. A smaller standard error denotes a more precise estimate. Asymmetry in the funnel plot may suggest potential publication bias or other small-study effects. Intervention Codes: AB, CT-guided percutaneous drainage combined with conventional antibiotic therapy CB, Conventional postural drainage combined with conventional antibiotic therapy DB, Modified postural drainage combined with conventional antibiotic therapy EB, Ultrasound-guided percutaneous drainage combined with conventional antibiotic therapy B, Conventional antibiotic therapy alone. ER, Effective Rate; LPS, Length of Hospital Stay.

To assess the potential impact of small-study effects on the aforementioned comparison results, we conducted tests for small-study effects on two indicators: the effective rate and the length of hospital stay. As shown in Figure 8, the *p*-values for Egger’s test corresponding to both indicators were > 0.05, indicating no statistical significance. This suggests a low probability of publication bias in these analyses. Visually, the left-right symmetry of the LPS studies appeared suboptimal, which may be attributed to heterogeneity fluctuations caused by the small number of studies and high heterogeneity between comparison groups, rather than indicating genuine publication bias.

### 3.8 Adverse events

Among the 23 included studies, five reported adverse events (16, 21, 23, 36, 37). For CT-guided percutaneous drainage combined with conventional antibiotic therapy (sample size = 29), 3 cases of pneumothorax occurred, resulting in an adverse event rate of 10.3%. For ultrasound-guided percutaneous drainage combined with conventional antibiotic therapy (sample size = 79), adverse events included 5 cases of pneumothorax, 1 case of pyopneumothorax, 3 cases of subcutaneous emphysema, and 1 case of hemoptysis, with an adverse event rate of 12.7%. For conventional postural drainage combined with conventional antibiotic therapy (sample size = 113), adverse events included 10 cases of pneumothorax, 5 cases of bronchopleural fistula with expectoration, 3 cases of empyema, 2 cases of pyopneumothorax, 4 cases of hemoptysis, 1 case of subcutaneous emphysema, 2 cases of chronic cough, 1 case of bronchitis, and 1 death, resulting in an adverse event rate of 25.7%. For conventional antibiotic therapy alone (sample size = 13), adverse events included 3 cases of empyema, 3 cases of chronic emphysema, and 2 cases of sepsis, with an adverse event rate of 61.5%. For modified postural drainage combined with conventional antibiotic therapy (sample size = 36), no adverse events (0 cases of chronic cough, bronchitis, or pneumothorax) were reported, resulting in an adverse event rate of 0%. As the vast majority of studies did not systematically report safety outcomes, and the reported event types and definitions were inconsistent, the above findings should be considered preliminary. Detailed adverse event specifics and corresponding interventions are presented in Supplementary Table 3.

## 4 Discussion

This study comprehensively synthesized existing evidence through a systematic review and utilized Bayesian network meta-analysis to overcome the limitations of traditional methods in multi-arm comparisons. The combination of both approaches offers unique advantages: the systematic review ensures transparency and reproducibility in evidence synthesis, while the Bayesian NMA provides optimal indirect comparison estimates when direct comparisons are lacking and offers a probabilistic ranking of the likelihood of each therapy being the best, which holds greater guiding significance for clinical decision-makers.

The results from the network meta-analysis indicate that in the probabilistic ranking analysis for the best total effective rate in treating pulmonary abscess, CT-guided percutaneous drainage combined with conventional antibiotic therapy is likely the most effective. Through precise CT localization, percutaneous catheter placement enables continuous negative-pressure aspiration of pus, allowing antibiotics to exert their maximum efficacy. The synergistic action of both significantly improves patients’ clinical symptoms and imaging findings, greatly enhancing the effective rate of pulmonary abscess treatment. CT provides high-resolution cross-sectional images, clearly displaying the exact location, size, morphology, depth, internal structure of the abscess, and its spatial relationship with surrounding structures (such as major blood vessels, heart, bronchi, bones, etc.) (38). This allows for planning an absolutely safe puncture trajectory, accurately avoiding important structures like blood vessels and bronchi, which can effectively prevent complications such as pneumothorax, hemorrhage, and bronchopleural fistula. Additionally, CT offers high spatial resolution and visualization of bone structures (39), unaffected by interference from intrapulmonary air or chest wall bones. It can clearly display deep-seated abscesses surrounded by aerated lung tissue and deep abscesses adjacent to or located behind bones (40). This is CT’s greatest advantage over ultrasound, making it suitable for complex pulmonary abscesses requiring highly precise puncture drainage, such as deep-seated abscesses, retro-osseous abscesses, and air-containing abscesses (41). However, it is noteworthy that CT-guided percutaneous drainage involves radiation exposure, conventional CT is not real-time, costs are higher, and the procedure requires transportation to and operation within the CT suite.

However, in the ranking of efficacy for length of hospital stay, time to cough resolution, and time to fever resolution, the regimen of ultrasound-guided percutaneous drainage combined with conventional antibiotic therapy demonstrated the greatest clinical benefit in all three. The core principle of ultrasound-guided percutaneous drainage is similar to that of CT guidance, both aiming to establish a drainage channel to evacuate pus, reduce the infectious burden, and promote healing. Unlike CT guidance, ultrasound-guided percutaneous drainage can be performed at the bedside, significantly reducing transportation risks for critically ill patients (42). It enables rapid decompression of the abscess cavity, re-expansion of compressed bronchi, and prompt alleviation of cough. The entire procedure is shorter in duration, allowing for an earlier start to treatment and a reduced length of hospital stay. Furthermore, post-drainage, there is a sharp decline in pyrogens within the abscess cavity, such as endotoxins, IL-1β, and TNF-α (43). Concurrently, the pH within the abscess cavity returns to normal, enhancing antibiotic efficacy, accelerating fever resolution, and improving the success rate of treatment. Ultrasound-guided percutaneous drainage is beneficial for achieving better and faster symptom relief (44). Additionally, ultrasound-guided imaging is real-time, allowing for dynamic adjustment of the needle tip direction to avoid accidental injury to the lung or blood vessels, ensuring accurate placement in a single attempt (45), and reducing the incidence of complications. It is particularly suitable for peripheral abscesses adjacent to the chest wall (46). In summary, ultrasound guidance offers advantages such as bedside operability, real-time monitoring, and shorter procedural time. It is also radiation-free and generally less costly compared to CT guidance.

A significant reduction in lesion size is a key objective imaging indicator for assessing the efficacy of pulmonary abscess treatment, directly reflecting the clearance of the abscess cavity and the repair of lung parenchyma. Network meta-analysis revealed that in the probabilistic ranking for the best outcome in terms of the number of lesion reduction, modified postural drainage combined with conventional antibiotic therapy was the most effective. Conventional postural drainage relies on a fixed position with the head lowered and the affected side elevated, depending primarily on gravity for natural drainage (47). However, patients with pulmonary abscess often have viscous pus and bronchial obstruction, leading to easy retention of purulent material. The cure of pulmonary abscess is not achieved by mere “evacuation.” While percutaneous drainage can rapidly aspirate pus, it is largely ineffective against gelatinous necrotic tissue adhering to the abscess wall (48). In contrast, modified postural drainage optimizes the drainage path by using CT to personalize the drainage angle. Combined with high-frequency chest wall percussion and controlled coughing, it disrupts pus viscosity and “dislodges” solid necrotic material into fragments that can be expelled—something a drainage catheter cannot achieve. Furthermore, when the patient assumes the position calculated by CT for drainage, a continuous fluid level gradient forms across the bronchi of the entire lung lobe. At this point, the active cough generates an airflow of 12 L/s, capable of “sucking” pus from fourth-level bronchi directly to the main bronchus within 0.5 s (49, 50). Leveraging hydrodynamic advantages, it rapidly expels pus with higher efficiency than percutaneous drainage. It is suitable for deep small abscesses (<5 cm) and patients at high surgical risk, while percutaneous drainage remains the preferred choice for peripheral abscesses > 5 cm (51). Additionally, modified postural drainage can be performed 4–6 times daily, continuously removing newly produced inflammatory exudates (52). This continuity is crucial for the steady reduction in lesion size.

This study compared the efficacy of different pus drainage procedures in the treatment of pulmonary abscess. Although relevant conclusions were drawn, the following limitations remain:

(1) Only five of the included studies reported adverse event data, resulting in insufficient statistical power and precluding meaningful network meta-analysis for safety outcomes. Therefore, the findings are far from conclusive. The safety data presented in this paper are merely descriptive summaries intended to reflect the reporting status in the existing literature and should only be considered preliminary.

(2) The vast majority of the included studies were conducted in China. This may lead to conclusions being more reliable for the Chinese population, requiring cautious extrapolation to other regions. However, as the pathophysiology of pulmonary abscess and the principles of treatment are universal, the conclusions may still offer valuable reference. Future validation through more international multicenter RCTs is warranted.

(3) The literature search strategy had language restrictions. To ensure feasibility, we only searched Chinese and English databases and limited inclusion to studies published in these two languages. This strategy undoubtedly missed relevant RCTs published in other languages. This may result in a less comprehensive evidence base and could introduce language bias, making the findings more reflective of clinical practices reported in Chinese and English literature, thereby further limiting the generalizability of the study’s findings to regions with different primary languages.

(4) The methodological quality of the included studies has limitations. Issues such as non-rigorous randomization, inadequate reporting of allocation concealment, and insufficient application of blinding for participants and outcome assessors in the included studies may introduce bias. Future validation through a substantial number of multicenter, large-sample, double-blind randomized controlled trials is still required.

## 5 Conclusion

Comprehensive analysis indicates that no single drainage method is superior across all outcomes. However, ultrasound-guided percutaneous drainage combined with antibiotics demonstrates the highest probability of improvement in key outcome measures, including time to fever resolution, length of hospital stay, and time to cough resolution, potentially showcasing comprehensive clinical benefit. CT-guided percutaneous drainage combined with conventional antibiotic therapy may be the most effective in terms of the effective rate, while modified postural drainage combined with conventional antibiotic therapy may be the most prominent in increasing the number of lesion reduction. Furthermore, conventional antibiotic therapy alone is inferior to pus drainage procedures combined with conventional antibiotics across multiple outcome measures and is associated with a higher incidence of adverse reactions. However, these findings can only be considered preliminary due to the limited number of available studies. This study provides an evidence-based foundation for the individualized selection of pus drainage procedures for pulmonary abscess. Future validation through a substantial number of multicenter, large-sample, double-blind randomized controlled trials is warranted.

## Data Availability

The original contributions presented in this study are included in this article/Supplementary material, further inquiries can be directed to the corresponding authors.
