# Supplementary material for: Effectiveness and safety of four drainage methods for lung abscess: a Bayesian network meta-analysis and systematic review
Source: Front Med (Lausanne). 2026 Jan 8;12:1735888. doi: 10.3389/fmed.2025.1735888 (PMC12823804; doi:10.3389/fmed.2025.1735888)
Supplement: Supplementary file 1 [file Table_1.docx]

Supplementary Table 1 PubMed Search Strategy Table

| **Databases:** | **PubMed** | |
| --- | --- | --- |
| **Search number** | **Search query** | **Search fields** |
| ****#1**** | ("Lung Abscess"[Mesh]) OR ("Pulmonary Abscess"[tiab]) OR ("Lung Abscesses"[tiab]) OR ("Lung Pyothorax"[tiab]) OR ("Pulmonary Suppuration"[tiab]) | All fields |
| ****#2**** | ("Drainage"[Mesh]) OR ("Therapeutic Drainage"[tiab]) OR ("Abscess Drainage"[tiab]) OR ("Percutaneous Drainage"[tiab]) OR ("Postural Drainage"[tiab]) | All fields |
| ****#3**** | ("CT-Guided Percutaneous Drainage"[tiab]) OR ("Computed Tomography Guided Drainage"[tiab]) OR ("Tomography, X-Ray Computed"[Mesh] AND "Drainage"[Mesh]) | All fields |
| ****#4**** | ("Ultrasound-Guided Drainage"[tiab]) OR ("Ultrasonography, Interventional"[Mesh]) OR ("Sonographically Guided Drainage"[tiab]) OR ("US-Guided Percutaneous Drainage"[tiab]) | All fields |
| ****#5**** | ("Postural Drainage"[tiab]) OR ("Positional Drainage"[tiab]) OR ("Bronchial Drainage"[tiab]) OR ("Gravity-Assisted Drainage"[tiab]) | All fields |
| ****#6**** | #2 OR #3 OR #4 OR #5 | All fields |
| ****#7**** | ("Randomized Controlled Trial"[pt]) OR ("Controlled Clinical Trial"[pt]) OR ("Randomized"[tiab]) OR ("Randomly"[tiab]) OR ("Placebo"[tiab]) OR ("Clinical Trial"[pt]) NOT ("Animals"[Mesh] NOT "Humans"[Mesh]) | All fields |
| ****#8**** | #1 AND #6 AND #7 | All fields |

Supplementary Table 2 GRADE Assessment Results Table

Note: RCT, Randomized Controlled Trial ER, Effective Rate LPS, Length of Hospital Stay TCR, Time to Cough Resolution TFR, Time to Fever Resolution NLR, Number of Lesion Reduction

| Outcome | Studies, n | Study design | Risk of bias | Inconsistency | Indirectness | Imprecision | publication bias | Certainty |
| --- | --- | --- | --- | --- | --- | --- | --- | --- |
| ER | 14 | RCT | Serious | Not serious | Not serious | Serious | Not serious | Low |
| LPS | 10 | RCT | Not serious | Not serious | Not serious | Serious | Not serious | Moderate |
| TCR | 9 | RCT | Serious | Not serious | Not serious | Not serious | Not serious | Moderate |
| TFR | 9 | RCT | Serious | Not serious | Not serious | Not serious | Not serious | Moderate |
| NLR | 9 | RCT | Serious | Not serious | Not serious | Serious | Not serious | Low |

Supplementary Table 3 Adverse Reaction Details and Interventions

Note: T, trial group; C, control group

| Author (Year) | Group Type | Intervention(s) | Adverse Reaction Type | | | | | Incidence of Complications |
| --- | --- | --- | --- | --- | --- | --- | --- | --- |
| Zhang HY 2023(16) | T | Modified postural drainage combined with conventional antibiotic therapy |  | | | | | 0 |
|  | C | Conventional drainage combined with conventional antibiotic therapy | Pneumothorax 2.78% | Bronchitis 2.78% | Chronic Cough 5.56% |  |  | 11.11% |
| Li WJ 2013(21) | T | Ultrasound-guided percutaneous drainage combined with conventional antibiotic therapy | Pneumothorax 6.67% |  |  |  |  | 6.67% |
|  | C | Conventional postural drainage combined with conventional antibiotic therapy. Identical anti-inflammatory therapy was administered to both groups. | Pneumothorax 20% | Bronchopleural fistula 6.67% | Empyema 6.67% |  |  | 33.33% |
| Zhang S 2010(23) | T | CT-guided abscess drainage combined with conventional antibiotic therapy. Systemic antibiotic use along with supportive and symptomatic treatments were consistent with those in the control group. | Pneumothorax 8.57% | Pyopneumothorax 2.86% | Subcutaneous emphysema 8.57% | Hemoptysis 2.86% |  | 22.86% |
|  | C | Conventional postural drainage combined with conventional antibiotic therapy. Antibiotic therapy primarily consisted of a combination of second- or third-generation cephalosporins and quinolones, with subsequent adjustments based on sputum culture and drug susceptibility results. | Pyopneumothorax 3.13% | Empyema 3.13% | Bronchopleural fistula 3.13% | Hemoptysis 9.38% | Death 3.13% | 21.88% |
| Wu ZY 2022(36) | T | In addition to the control therapy, the patients underwent ultrasound-guided percutaneous drainage. | Pyopneumothorax 2.63% | Pneumothorax 5.26% | Subcutaneous emphysema 7.89% | Hemoptysis 2.63% | Bronchopleural fistula 0 | 18.42% |
|  | C | Conventional postural drainage combined with conventional antibiotic therapy. A combined treatment regimen was adopted, primarily consisting of second- or third-generation cephalosporins in conjunction with quinolones, supplemented by aerosol inhalation and postural drainage. | Pyopneumothorax 2.63% | Pneumothorax 7.89% | Subcutaneous emphysema 2.63% | Hemoptysis 2.63% | Bronchopleural fistula 5.26% | 21.05% |
| Mohamed A S 2014(37) | T | Ultrasound-guided percutaneous drainage combined with conventional antibiotic therapy.  These empirical antibiotics were remodulated after the result of aspirates culture and sensitivity. | Pneumothorax 7.7% | Incomplete aspiration 15.4% |  |  |  | 23.08% |
|  | C | Systemic empirical antibiotic therapy, with subsequent adjustment of the treatment regimen based on the results of sputum culture and sensitivity testing. | Empyema 23.2% | Chronic abscess 23.2% | Septicemia 15.4% |  |  | 61.54% |
